# Functional Anatomy of Writing with the Dominant Hand

**DOI:** 10.1371/journal.pone.0067931

**Published:** 2013-07-02

**Authors:** Silvina G. Horovitz, Cecile Gallea, Muslimah ‘Ali Najee-ullah, Mark Hallett

**Affiliations:** 1 Human Motor Control Section, Medical Neurology Branch, National Institute of Neurological Disorders and Stroke, National Institutes of Health, Bethesda, Maryland, United States of America; 2 Centre de Neuro-imagerie de Recherche, Hôpital Pitié-Salpêtrière, Institut National de la Santé et de la Recherche Médicale, Paris, France; University Medical Center Groningen UMCG, The Netherlands

## Abstract

While writing performed by any body part is similar in style, indicating a common program, writing with the dominant hand is particularly skilled. We hypothesized that this skill utilizes a special motor network supplementing the motor equivalence areas. Using functional magnetic resonance imaging in 13 normal subjects, we studied nine conditions: writing, zigzagging and tapping, each with the right hand, left hand and right foot. We identified brain regions activated with the right (dominant) hand writing task, exceeding the activation common to right-hand use and the writing program, both identified without right-hand writing itself. Right-hand writing significantly differed from the other tasks. First, we observed stronger activations in the left dorsal prefrontal cortex, left intraparietal sulcus and right cerebellum. Second, the left anterior putamen was required to initiate all the tested tasks, but only showed sustained activation during the right-hand writing condition. Lastly, an exploratory analysis showed clusters in the left ventral premotor cortex and inferior and superior parietal cortices were only significantly active for right-hand writing. The increased activation with right-hand writing cannot be ascribed to increased effort, since this is a well-practiced task much easier to perform than some of the other tasks studied. Because parietal-premotor connections code for particular skills, it would seem that the parietal and premotor regions, together with basal ganglia-sustained activation likely underlie the special skill of handwriting with the dominant hand.

## Introduction

Writing is an example of a fine motor skill. From a kinematic prospective, it requires fine-tuning of finger position to manipulate a familiar object. It involves sensory-motor integration to position the limb in space as well as access to language content. The ability to perform a skilled movement with different effectors while keeping common movement kinematics led to the concept of motor equivalence [1], [2]. The motor equivalence theory supports the existence of a writing motor program, which stores the movement characteristics in a higher order area than the primary motor cortex and would be accessible by each effector (a hand, a foot or the mouth). The writing motor program is an abstract representation of movements with invariant features pertaining to the order of event sequences and timing that can be executed by any effector [3]. Areas for “writing” and for “effector” would be coupled to allow writing by any effector.

The functional neuroanatomy of writing was perhaps first assessed by Exner [4] who described an area in the middle frontal gyrus superior to Broca’s area that organized the motor behavior necessary for fluent writing. Several functional magnetic resonance imaging (fMRI) neuroimaging studies aimed to isolate brain areas involved in the writing motor program [5], [6], [7], [8]. These studies found that left parietal and premotor areas (BA6) were active when writing with the dominant hand. Rijntjes et al. [9] identified the anterior aspect of the dorsal premotor cortex (PMd) as the location where the writing program resides and would be retrieved by each limb to perform a writing task, as indicated by the motor equivalence theory. The posterior part of PMd displayed activations more specific to each limb. PMd was also identified as an underactive region during motor planning with the goal of writing, irrespective of its execution, in writer’s cramp patients [10].

The motor equivalence program explains the substrate of how to perform the same task with different limbs. Why, then, does a right-handed subject prefer writing with the right hand and is more skillful at it than when writing with any other limb? Writing with the dominant hand is a task learned over time. The preference and skill a person has for an over-learned task should have some neurological substrate, which should go beyond motor equivalence. This can potentially explain disorders such as writer’s cramp, a type of task-specific focal hand dystonia (FHD), in which patients lose the ability to write with their dominant hand, while still being able to perform other motor tasks with the dominant hand and write with another limb [11]. Thus, we equate the specific neurological substrate as an indication of task specificity for the skillful task. Alternatively, dominant hand writing could rely on better connections between brain areas involved in the writing motor program and brain areas controlling the dominant hand rather than on a different anatomical substrate. Here, we attempt to understand the functioning of brain networks during skillful performance using dominant handwriting as a probe.

To this end, we conducted an fMRI study on healthy volunteers and included three effectors (right hand, left hand, right foot) and three tasks (writing, zigzagging, tapping) using an experimental paradigm similar to that of Rijntjes et al. [9] but with a different goal. Rijntjes et al. looked for the anatomical representation of writing, irrespective of the limb used, as explained by the motor equivalence theory. We aimed to understand the neurological imprint of skillful tasks performance, such as writing with the dominant hand. We expected this skillful task would recruit a brain network beyond the activation needed for writing with other effectors, or in using the dominant hand for other tasks.

## Methods

### Subjects

Thirteen healthy individuals (mean age 38.6±12.3 years, 6 women) participated in the study. All participants were right-handed according to the Edinburgh Handedness Inventory [12]. Subjects did not have any neurologic or psychiatric disorder, or history of alcohol or substance abuse. All individuals were screened for MRI safety and were compensated for their participation.

#### Ethics statement

The NIH Institutional Review Board approved the study and all subjects gave written informed consent.

### Experimental Tasks and Design

Subjects had a digitizing tablet (fMRITouchscreen, Mag Design and Engineering, Redwood City, CA) placed close to either their right or left hand or their right foot. For each run, a stylus was held in the right hand (RH) or the left hand (LH) or fixed between the first and second toes of the right foot (RF) with an elastic bandage. The subjects held the stylus with the corresponding limb before the run started to avoid confounds from reaching and grasping the object when performing the task. The subjects performed three movement tasks: writing (W), tapping (T), and zigzagging (Z) with each limb. For the writing task, they wrote the sentence: THE QUICK BROWN FOX JUMPS OVER THE LAZY DOG, in all capital, block letters. While we did not expect differences based on the individual graphemes or the use of block letters, this sentence provides a uniform task for all subjects with a variety of shapes. For the tapping task, subjects tapped at a slow and steady pace, approximately one tap per second, without counting. The writing and zigzagging tasks were performed at approximately one move per second. Prior to the fMRI, all participants practiced the nine tasks until they could perform them proficiently and with minimal movement of the proximal limbs.

The study was carried out using a block design with all conditions lasting 20 sec each. Each movement condition alternated with a rest condition to allow the signal to return to baseline with six repetitions of a movement condition per run to reach sufficient statistical power [13]. During the rest condition, the subject relaxed the limb and stayed still. Each task was performed by each limb in a different run, for a total of nine runs (3 tasks×3 limbs). Each run lasted approximately 4 min 30 sec. At the beginning of each run, the subject was told which task to perform with which limb; the stylus was held with the corresponding limb. During the run, the subject received the verbal cues “start” to indicate the beginning of the task and “relax” to indicate the beginning of the rest condition. All motor tasks were internally driven, with no visual inputs, at the pace stipulated during the training session. Each run began with a rest condition. The order of runs for each of the nine tasks was randomized between subjects.

### Data Acquisition and Preprocessing

Participants’ heads were stabilized in the receiving/head coil by firm foam pads to avoid movement. Both arms were in the supine position and supported by foam pads and cushions to provide relaxation of proximal limb muscles. Subjects were scanned in a 3.0 T General Electric MRI scanner with an 8-channel head coil and an echo planar imaging (EPI) sequence with the following parameters: repetition time (TR) 1.75 sec, echo time (TE) 30 msec, flip angle (FA) 90°, matrix 64×64 mm^2^, 32 interleaved slices 4.0 mm thick, including an 0.5-mm gap, and a field of view (FOV) 22 cm, yielding a resolution of 3.5×3.5×4.0 mm^3^.

The first four EPI volumes were discarded to allow magnetization to reach steady state. Imaging data were pre-processed using SPM5 [14], [15]. Data were adjusted for slice timing, realigned to the first image of the first run (motion corrected), normalized to the Montreal Neurological Institute template, and smoothed using an 8-mm Gaussian kernel. Head motion parameters were monitored online during scanning; subjects moving their heads more than 2 mm repeated the run. Subjects’ performances were monitored for compliance with instructions during each run by looking at the tablet data. Runs were restarted if the subject did not follow the verbal command to start the task, did not perform the task properly, or had excessive head motion. Behavior was observed for compliance, but quantitative behavioral data were not recorded.

### Data Analysis

A two-level analysis was performed using SPM5 and SPM8 [14]. In the first level, we created within-subject contrasts of each task condition versus rest. We used a general linear model (GLM) to estimate the amplitude of the blood oxygen level-dependent (BOLD) signal comparing task-specific activity with rest (individual t-test). In the second level, we entered these contrasts in a group analysis, treating each subject as a random effect. Here, we used a 3×3 within-subjects ANOVA model with individual measurements (13 contrast images) sorted in a matrix of nine cells. We modeled the data in terms of the two main effects (task and limb) and one interaction (task×limb), setting the significance at p<0.05, family-wise error (FWE) corrected.

We performed subsequent analyses to answer three questions. We first asked, “How does the writing program differ from the other programs?” To answer this question, we computed the differences (t-test) between the levels of factor *Task* (writing versus tapping; zigzagging versus tapping), averaging over the levels of the factor *Limb*. Then, we masked the contrast ‘writing versus tapping’ with the contrast ‘zigzagging versus tapping’ (exclusive mask thresholded at p<0.001). The exclusive masking allowed isolating those brain areas that were only significantly activated during writing but not during zigzagging. This resulted in a map of task-specific effect for writing in terms of amplitude of the BOLD signal. This contrast, performed in SPM5, was FWE-corrected at p = 0.05. The analysis shows the areas used for writing, irrespective of the limb used, thus indicating the motor equivalence program for writing.

To answer the second question, “Does right-hand writing activation show task specificity?”, we looked at brain areas significantly more active for right-hand writing (RHw) than for any of the other tasks. In logic, this is defined as an AND of the t-test between RHw and each of the other conditions. Two procedures were implemented: a conjunction and an exclusive masking. In the first procedure, we implemented the conjunction significance to t_8,118_ = 2.37 in SPM (one-tailed Dunnett test, with RHw as the reference and the other eight tasks as the treatments). We extracted each task’s time course from a sphere having a 3-mm radius centered at the activation peaks. For each region, the time course of RHw was used as the control task in a repeated measures one-way ANOVA, with Dunnett correction for multiple comparisons. Testing was performed in Prism 6.0b (GraphPad Software, Inc). This analysis indicated voxels that were significantly more active for RHw than for each and all the other tasks. However, the resulting areas could also be active for other tasks besides RHw. Therefore, we performed a second procedure as an exploratory analysis to isolate regions only active for RHw. We defined regions of interests (ROIs) in the areas where RHw task was highly significant (p<0.001, FWE corrected) and masked out any voxel that had significant activation (p<0.05 FWE corrected) in any other task. Stringent and conservative thresholds were selected to decrease the chances of false positive results in each contrast and in the masking procedure. For each ROI, we extracted each task’s time course from the activation peaks. For each region, the time course of RHw was used as control task in a repeated measures one-way ANOVA, with Dunnett correction for multiple comparisons. Testing was performed in Prism 6.0b (GraphPad Software, Inc).

To answer the third question “Does dominant hand writing rely on better connections between brain areas involved in the writing motor program and brain areas involved in controlling the dominant hand?”, we carried out a functional connectivity analysis using a psychophysiological interaction model [16]. PPI analyses identified the areas in which the degree of coupling with the index region was modulated specifically by the RHw task compared to each of the other tasks. The index area included the cluster showing the highest T value for the writing motor program (isolated in the first question). The mean corrected and high-pass-filtered time series in the index area were obtained on a subject-by-subject basis by extracting the first principal component from all time series. PPI regressors were computed as the element-by-element product of the deconvolved extracted time series and a vector coding for the main effect of task. Each PPI regressor was mean corrected to remove subject-specific effects and convolved by the canonical HRF to account for a possible hemodynamic lag. For each subject, the PPI regressor, task regressor, and extracted time series were entered in a first-level model. At the individual level, PPI analysis was carried out for each motor task versus rest. At the group level, the 9 individual PPI t-contrasts were submitted for a group analysis in a full factorial design (3×3 ANOVA (task×limb)). We implemented the conjunction significance to t_8,118_ = 2.37 in SPM (one-tailed Dunnett test) to look at voxels that were significantly more correlated with the writing motor program area during RHw than for each and all the other tasks.

## Results

### Behavior

Performance was monitored as subjects performed the tasks. Most subjects completed the nine runs without difficulty. Of 13 subjects, one performed all nine runs correctly at the first trial. Six subjects had to restart one run. Two subjects each repeated two runs and two others each repeated three runs. One subject had to restart six runs and another subject had problems in each run, for a total of 25 repetitions. There was no correlation between performance and individual task-effector combination. Imaging analysis was performed only using the runs with adequate performance.

### Main Effects

**Figure 1:**
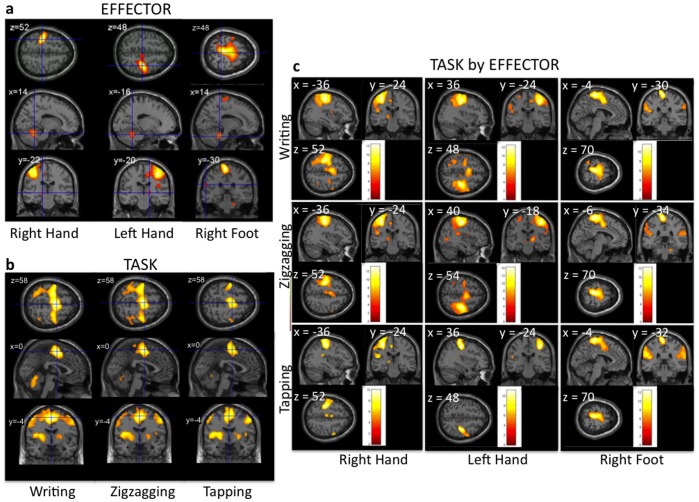
Main effects. Brain areas significantly activated for the main effects of each effector (**a**) and the main effect of each task (**b**) and for each of the nine conditions (**c**) (p<0.05, FWE corrected).

The ANOVA showed significance for main effect of both *Task* and *Limb* (F = 15.02, p<0.05, FWE corrected), and for the interaction between *Task* and *Limb* (F = 9.38, p<0.05, FWE corrected). Figure 1 displays the statistical maps for main effects (Fig. 1a–b) and for the nine conditions (Fig. 1c).

#### Main effect of limb

Contralateral and ipsilateral activations were relative to the side of the moving limb and followed somatotopic representation. Performing the tasks with each ‘limb’ engaged the motor circuitry, including the contralateral primary motor cortex and ipsilateral cerebellum, following the somatotopic representation of the limb used. Activation maps for the RH, LH, and RF are seen in Fig. 1a. Areas involved for each limb used are shown in Table 1.

**Table 1:** Effect of LIMB: Regions activated for the right hand, left hand, and right foot.

Region	*x*	*y*	*z*	Peakt-value	Cluster size
					(mm^3^)
**Right hand**					
Left motor and sensorimotor cortex	−36	−26	52	16.61	18984
Right cerebellum (declive/culmen)	14	−52	−24	8.81	5912
Left thalamus (ventral posterior	−14	22	−2	5.54	112
medial nucleus)					
Left premotor, SMA	−6	−24	48	5.32	96
**Left hand**					
Right motor and sensorimotor cortex	38	−24	48	21.08	35920
Left cerebellum (declive/culmen)	−16	−52	−26	10.59	3888
Right insula	42	−22	20	6.68	784
Right thalamus (ventral posterior	16	−20	0	7.03	552
medial nucleus)					
Right medial frontal gyrus	6	50	20	5.14	152
**Right foot**					
Left premotor, SMA	−4	−32	68	17.85	20976
Right cerebellum (dentate/culmen)	14	−38	−32	10.39	4432
Left insula/putamen	−30	−24	14	6.25	872
Left postcentral gyrus/	−60	−28	22	5.71	704
Supramarginal gyrus					
Left middle cingulate cortex	−8	−8	44	6.49	488

#### Main effect of task

The tasks activated similar regions, but with differences in detail and magnitude. Common regions included bilateral frontal areas (medial frontal gyri, precentral gyri, rostral supplementary motor area (SMA)), bilateral inferior parietal areas, left supramarginal gyrus, left paracentral areas and bilateral cerebellum (declive) (Fig. 1b). Coordinates and statistics of the significantly activated clusters are shown in Table 2. See below for more details with regard to just the writing task.

**Table 2:** Effect of TASK: Regions activated for writing, zigzagging, and tapping.

Region	*x*	*y*	*z*	Peakt-value	Cluster size
					(mm^3^)
**Writing**					
Bilateral frontal cortex (SMA,	−4	−8	56	11.27	118360
paracentral and precentral cortex)					
Right cerebellum (culmen/declive)	28	−52	−32	6.63	11208
Right inferior parietal cortex	36	−44	44	6.11	10592
Left cerebellum (culmen/declive)	−20	−64	−28	8.32	3400
Right lentiform nucleus	14	−14	2	5.39	2880
**Zigzagging**					
Bilateral frontal cortex (SMA,	−2	−8	56	11.24	137740
paracentral and precentral cortex)					
Right cerebellum (culmen/declive)	28	−52	−32	7.41	7280
Left cerebellum (culmen/declive)	−22	−62	−28	7.69	3240
Right lentiform nucleus	14	−16	2	5.77	3200
Right middle temporal occipital gyrus	42	−68	2	6.05	1792
**Tapping**					
Left inferior parietal cortex	−48	−26	18	10.46	69488
Right precentral, insula	54	0	48	8.90	24320
Right inferior parietal cortex	62	−36	18	8.17	13936
Right inferior middle frontal gyrus	42	42	14	5.62	1096
Left middle temporal occipital gyrus	−52	−72	−12	5.38	520
Right cerebellum (declive)	26	−64	−26	5.04	128

#### Task- limb interaction

The interaction of task and limb was significant (F = 9.38, p<0.05; FWE corrected) in a cluster in the right post-central gyrus (MNI coordinates: [52, −18, 54]). This interaction was driven by left-hand activation, which was significant in this region for writing and zigzagging and, to a lesser extent, tapping. This region showed no significant activations for either RF or RH. However, for the RF, activation level for tapping was larger than zigzagging or writing. This interaction might be explained by a larger movement amplitude during tapping (flexion/extension of the ankle) than during zigzagging or writing (adduction/abduction of the ankle); it is not of major interest and will not be discussed further.

### The Motor Equivalence Program for Writing

**Figure 2:**
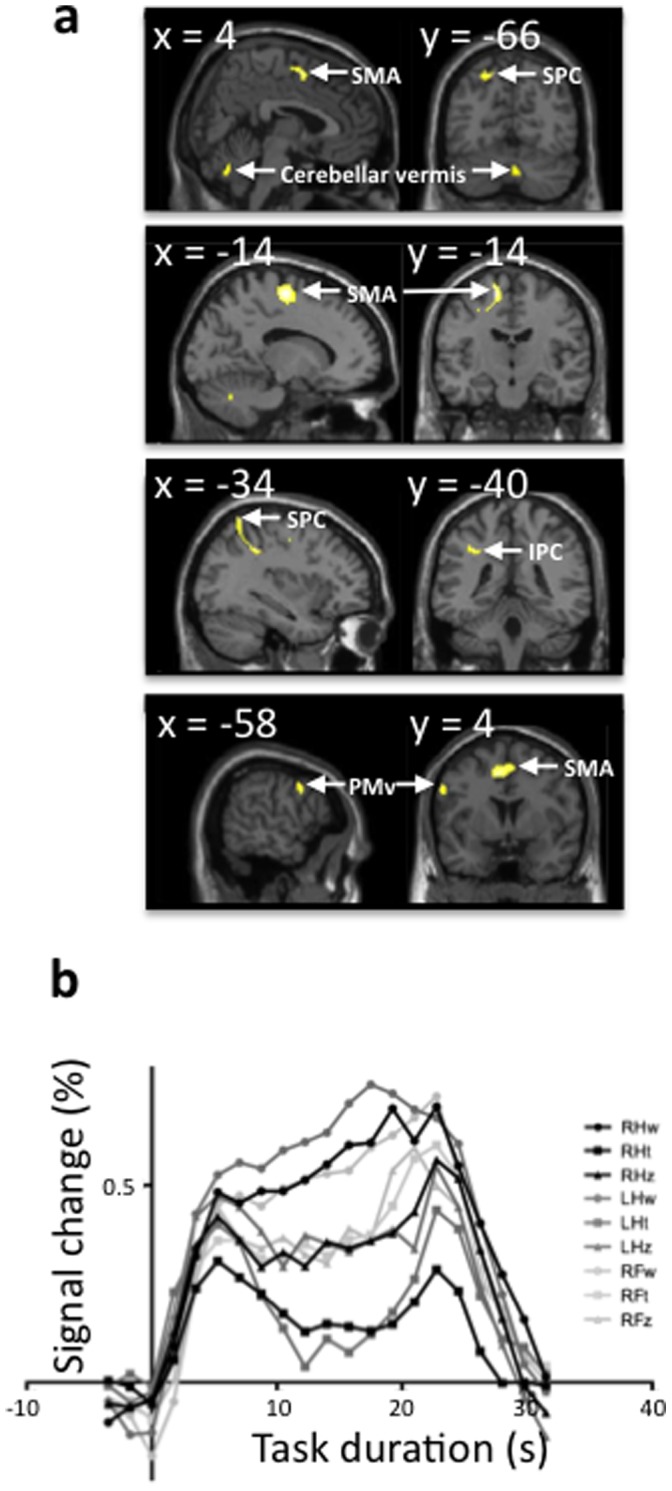
Exclusive activation for writing. Areas significantly activated for writing versus tapping (p<0.05, FWE corrected) with exclusive mask for zigzagging-tapping (p<0.001 uncorrected). **a).** Sagittal and coronal view of clusters (SMA = supplementary motor area; PMd = dorsal premotor cortex; SPC = superior parietal cortex; IPC = inferior parietal cortex; PMv = ventral premotor cortex). **b**). Time courses extracted from SMA (sphere centered on [−8 2 50]; see detailed statistics in Table 3) during the nine tasks (RH = right hand; LH = left hand; RF = right foot; w = writing; t = tapping; z = zigzagging).

Writing produced the largest BOLD amplitude change of the three tasks, while the smallest was finger tapping. To determine how the writing program differed from the other programs, we compared writing and zigzagging to finger tapping to eliminate the effect of motor execution and to observe the motor program. Furthermore, to focus on the writing motor program, we masked the ‘writing’ with ‘zigzagging’ (Fig. 2). The resulting contrast map included left cortical area activations in the SMA, Exner’s area, motor precentral cortex, a cluster extending in the superior and dorsal inferior aspects of the intraparietal sulcus, and a bilateral cluster in the cerebellum (vermis, lobules 6 and 8) (Fig. 2a, Table 3). The time courses extracted from the SMA during the nine tasks (Fig. 2b) showed a larger amplitude of the BOLD signal during the three writing tasks.

**Table 3:** Exclusive activations for writing.

Region	*x*	*y*	*z*	Peakt-value	Cluster size
					(mm^3^)
Left SMA/premotor cortex	−14−8	−142	6250	6.77	4136
Left superior/inferior parietal cortex	−34	−40	40	5.88	1480
Left ventral premotor cortex	−58	4	36	5.45	200
Bilat cerebellum (vermis, lobules 6/8)	4	−66	−34	4.84	248

(Regions activated for writing–tapping (exclusive mask zigzagging–tapping [P<0.001]); P<0.05, FWE corrected. Bilat = bilateral.

### Distinct Right-hand Writing Activations

**Figure 3:**
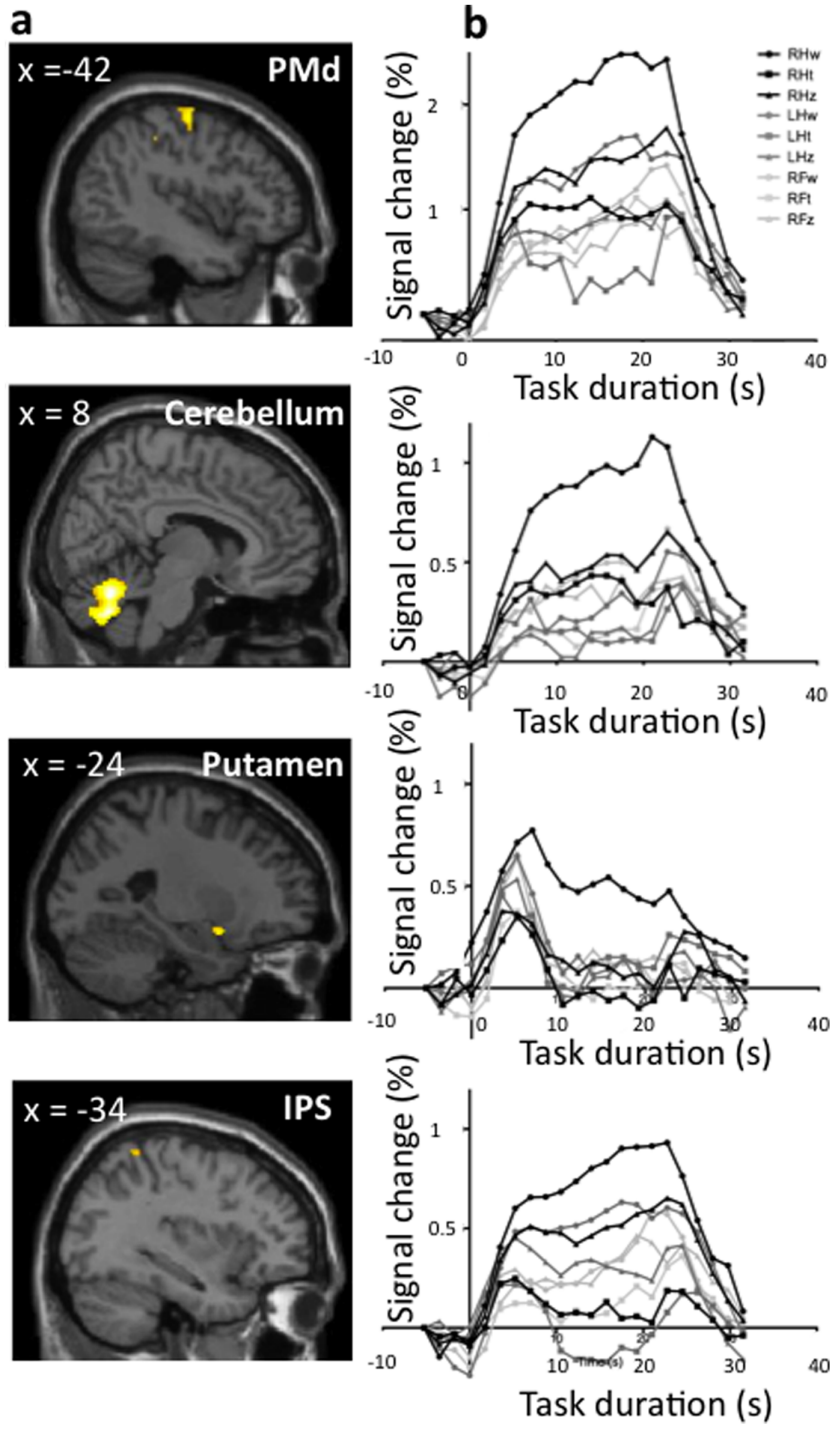
Distinct right-hand writing activations – significantly larger BOLD response. Conjunction of the differences between right-hand writing and each of the other tasks, thresholded at t<2.37, (Dunnett test one tailed). **a**) Cluster of activations, displayed over sagittal anatomical images. **b**) BOLD time courses of each task, for each of the clusters shown in **a**). PMd = dorsal premotor cortex; IPS = intraparietal sulcus.

RHw showed more activations than any other task (p<0.05, Dunnett corrected) in the left motor and dorsal premotor cortex (PMd), the left superior parietal cortex (SPC), a small cluster in the anterior putamen, and right cerebellum, more precisely in lobules 6 and 8 involved in sensorimotor tasks [17] (Table 4). Figure 3a shows the areas with a significantly larger amplitude of the BOLD signal for RHw than for any of the other eight tasks.

**Table 4:** Activations for RHw that are greater than any other task.

Region	*x*	*y*	*z*	Peak t-value	Cluster size
					(mm^3^)
Right cerebellum (dentate, lobules 6 and 8)	8	−62	−24	3.86	4184
Left motor and dorsal premotor	−46	−12	62	2.90	248
Left anterior putamen	−24	8	−14	2.58	56
Left superior parietal cortex, IPS	−32	−48	64	2.37	10

(Dunnett test, t>2.37).

IPS = intraparietal sulcus.

Time courses from the clusters significantly more active for RHw than the other tasks demonstrated different regional patterns of activations (see Fig. 3b). PMd had the largest BOLD increase of all the regions, but was significantly active for other conditions besides RHw; this region has strong activations for LHw and RHz. A similar pattern is observed in SPC, but with lower activation levels. The cluster in the cerebellum also showed a low level of activation for other tasks performed with the RF and RH. The left anterior putamen was recruited for all tasks to initiate the task, but only sustained a high activation level during task performance for RHw. To check for the overall activity level, we also analyzed the time course of the SMA, a region involved in writing regardless of the effector. We found it was similar for RH, LF and RF writing (Fig. 2c).

**Figure 4:**
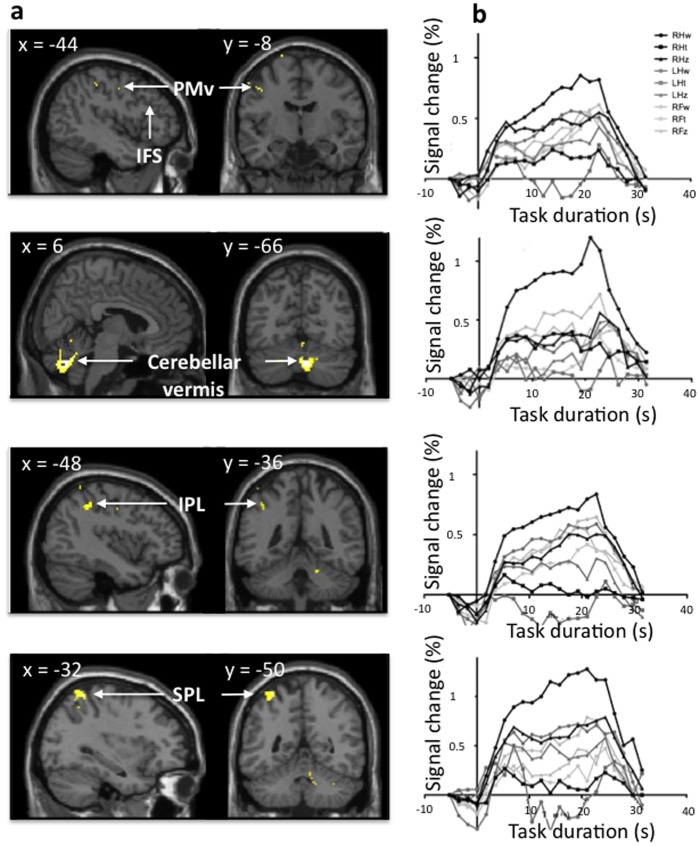
Distinct right-hand writing activations – unique significant BOLD response. Right-hand writing activations, thresholded at p<0.001 FWE corrected, and masked by each of the other tasks, thresholded at p<0.05 FWE corrected. **a**) Cluster of activations, displayed over coronal and sagittal anatomical images. **b**) BOLD time courses of each task, for each of the clusters shown in **a**). PMv = ventral premotor cortex; IFS = inferior frontal sulcus; IPL = inferior parietal lobule; SPL = superior parietal lobule.

The exploratory analysis identified areas uniquely activated for RHw in the left ventral premotor cortex, left anterior putamen, left inferior and superior parietal cortices, and right cerebellum (Fig. 4 and Table 5). Areas in the right cerebellum and superior parietal cortex overlapped for the two analyses.

**Table 5:** Activations unique to RHw.

Region	*x*	*y*	*z*	Peakt-value	Cluster size
					(mm^3^)
Right cerebellum (lobules 6 and 8)	6	−66	−34	8.39	2680
Left superior parietal cortex	−32	−50	66	6.43	784
Left motor and ventral premotor	−44	−8	44	5.82	128
Left anterior putamen	−22	8	2	5.68	88
Left inferior parietal cortex	−48	−36	44	5.67	144

(RHw : p<0.001 FWE; all other tasks p<0.05 FWE ).

### Functional Connectivity between Writing Motor Program Area and Right Hand Area

The index area used in the PPI analysis was the cluster including the SMA (Table 3). The main effect of limb showed an increase of functional connectivity between SMA and the hand area of the left primary motor cortex (MNI coordinates [x y z] = −34 −24 54, F = 7.55, p<0.001 uncorrected). The conjunction analysis did not reveal any difference of functional connectivity within the SMA network during RHw compared to all the other tasks.

## Discussion

RHw, our model for a skillful over-learned task, showed both higher activation and recruited a larger brain network than writing with other limbs or performing simpler but a less practiced task with any limb. Additionally, a motor equivalence area such as the SMA was equally activated during writing tasks and did not show any specific increase of functional connectivity with the hand area of the primary motor cortex during RHw compared to other tasks. We confirmed that both PMd and SMA had a pivotal role in writing (Fig. 2), and that PMd had higher activation especially during RHw compared to all other tasks (Fig. 3). Similar results were seen for the intraparietal sulcus, putamen and cerebellum. The left anterior putamen had a unique pattern of activation: while all tasks recruited this region at the onset of the task, only RHw required sustained increased activity for the full duration of the task. Our exploratory analysis showed a second premotor-parietal network, in the ventral region, that was only significantly activated during RHw (Fig. 4).

Our study differs from other recent work in how we separated the different components of a complex movement. Glover et al. [18] studied the different networks involved in planning and controlling a reach-grasp movement; Delnooz et al. [19] compared reaching to grasping a pencil for shaping or writing. Our task selection attempted to control for reaching and grasping by having the subjects hold the stylus throughout each scan run. Task selection also tried to control for planning, since each of the three effectors performed each of the three tasks. Thus, our study identified the areas of the writing program, as previously identified in other imaging studies [9], [20], in agreement with the motor equivalence theory [1], [21]. Our study also identified a second premotor-parietal network, with anterior putamen support, that supplements the areas of the motor equivalence theory during writing with the dominant task. Our study surveyed the time courses of the areas of interest, finding sustained putaminal activation only for the RHw.

### The Writing Motor Program

Writing recruited a larger set of brain areas than either zigzagging or tapping. Our results reproduced the findings of Rijntjes et al. [9]. We found that activity in the SMA, left premotor areas, including Exner’s area, identified as the handwriting center [4], clusters in the parietal cortex, including the superior and inferior cortices, and vermis were specific to writing regardless of the effector used. The premotor areas seen here include PMd, an area associated with the transformation of the motor program into the effector, possibly anchor of the ‘motor equivalence’ [21]. TMS over PMd is known to affect the coupling between grasping and lifting. In our study, since subjects had the stylus in the limb, neither gross grasping nor lifting were studied [22]. However, fine adjustments of the grasp are needed for writing, while much less is required for zigzagging and even less for tapping. Indeed, in agreement with previous work [9], we found PMd was active in all writing tasks, supporting its role as a key area for the writing program.

PMd projects to the superior part of the parietal cortex, where sensorimotor integration occurs, and the motor cortex, where the movement is executed [23]. The cluster in the superior parietal area included the regions seen active for narrative and simple graphemes, but not for motor response nor language tasks [20], suggesting the drive for activation seen in these areas is not the language content but rather its motor expression.

Electrocortical mapping of writing tasks also supports our findings. Roux et al. [8] showed that partial removal of Exner’s area affects handwriting and that this area is active while writing with either hand. Our finding that the SMA had a major role in the writing motor program is supported by the anatomical organization of the SMA and its involvement in coordinating complex motor subroutines. The SMA contains a cortical somatotopy of the limbs [24], [25], but the body part representations overlap [23], [26]. This overlap might enable coordination of movements of different body parts [27], [28]. The SMA also contains a representation of complex movements [29], [30], and the same SMA neurons can discharge during well-learned movements regardless of the effector limb [31], [32], [33]. The observed writing-specific SMA activation is likely independent of the language component of writing. Language tasks preferentially activate the pre-SMA, superior temporal sulcus, and Broca’s area [34]. These areas were not activated in our tasks. Instead, the other areas activated for writing are highly connected with the SMA.

At the cerebellar level, the writing program recruited the vermis, dentate and lobules 6 and 8. Since RHw was included in the *writing* condition, it is likely that, at least in part the activations are driven by the RHw task, as discussed below.

### Distinct Right-hand Writing Activations

For right-handed subjects, writing with the LH or RF are less practiced tasks, and therefore are expected to be more difficult. Writing with the RH is the most over-learned writing task we tested; hence, it should not be the most difficult or effortful one, but the one that is the most efficiently performed. It is likely that subjects had much refined control of the fingers of their RH. However, RHw recruits a larger network when compared with the other tested task. Additionally, the motor equivalence areas were equally activated during all writing tasks and had equivalent functional connectivity with the right-hand motor area for all tested tasks. Therefore, dominant hand writing did not rely on better connections between brain areas involved in the writing motor program and brain areas involved in controlling the dominant hand. Thus, the additional network involved in RHw would support the efficient execution of overlearned sensorimotor skills. We speculate these brain areas, or their connections, encode for task specificity.

#### Premotor areas

PMd had a significantly stronger activation for RHw than for any other task. This dorsal pathway is related to motor kinematics and thus, present in movement planning and executing, regardless of the effector used; moreover, it has been characterized as *acting on* an object [35]. PMd participates in space encoding and object manipulation needed, to a lesser extent than for writing, for the zigzagging task. Our exploratory analysis showed a more ventral area of the premotor cortex (PMv) uniquely and strongly active for RHw. The border between PMd and PMv is not strictly delimited [36], [37] and PMv is frequently labeled as PMd in imaging studies [38]. An fMRI meta-analysis showed that the coordinates obtained in the present study are within an area of overlap between the two regions [39]. Monkey studies [37], [40], [41], [42], [43], [44], [45] indicated that the division between PMd and PMv was roughly the projection of the principal sulcus virtually crossing the arcuate and central sulci; this boundary would correspond to the virtual projection of the superior frontal sulcus on the precentral gyrus in human functional MRI studies [38], [46]. Since the part of the premotor area uniquely active for RHw was located lateral to this boundary (Fig. 4), we labeled it PMv. The ventral pathway is related to imagery and cognitive aspects of a movement, in particular, with the concept of *acting with* the object, where the schema for the purposeful use of an object is learned over time [35], thus matching only the RHw task, from the set we tested. The imaging and cognitive aspects of the writing condition for writing differ from the other two tasks, but are similar for all writing, regardless of the limb used. Transcranial magnetic stimulation (TMS) studies showed that sequential recruitment of intrinsic hand muscles is affected by a lesion in the contralateral PMv, but only the left PMv activated for RHw; the contralateral homologous area was not active for LHw.

#### Parietal areas

The parietal components from the dorsal and ventral premotor pathways also show distinct activations for the RHw condition. The intraparietal sulcus (IPS, Fig. 3) with strongest activation for the RHw, relates to the dorsal pathway. The IPS was suggested to be the equivalent of the monkey’s anterior intraparietal area (AIP). In monkeys, complex object manipulation is orchestrated in the ventral premotor area F5, which receives input from AIP. The neurons in these areas activate when using objects for which the animals have trained [47]. In turn, AIP is connected to the superior parietal lobule and F5 projects to the motor cortex. Our exploratory analysis showed unique activation in the human equivalent of these cortical areas, suggesting that these areas may support the use of a known object to perform a skillful task with a known object such as writing with a pen held by the dominant hand.

#### Cerebellum

The dentate and vermis were involved in all tasks performed by any of the three limbs, although the clusters were significantly larger and unique for RHw. The dentate nucleus is involved in movement generation and control, and projects to several areas of the cerebellar-thalamo-cortical circuit from its dorsal aspect. It is also involved in visuospatial control, at its ventral part [48]. Parts of the cerebellar right lobules 6 and 8 were activated only during RHw. These lobules contain a sensorimotor representation (somatotopy) of the ipsilateral hand [17]. Moreover, right cerebellar anomalies were observed in imaging studies of apraxic agraphia, a disorder affecting the ability to write letters [49]. The role of the cerebellum in high-order cognitive tasks is being increasingly documented [50], [51]. Our findings, together with evidence from the literature, suggest that cerebellar areas could be part of the specificity network for an over-learned task with highly demanding skills, such as writing with the dominant hand. This also agrees with the greater evidence of the influence of the premotor areas on cerebellar activity via the cortico-ponto-cerebellar pathway [52].

#### Subcortical involvement

The most anterior part of the left putamen showed a significantly higher activation level for RHw. While the putamen is involved in initiating all motor tasks in the present study, continuous engagement was seen only during RHw. The anterior portion of this middle part of the putamen receives inputs from premotor cortices [53] where the executive and associative thalamo-cortico-striatal loops overlap. Activation switches from rostral to caudal regions of the putamen when the level of task automatization increases [54]. Our results are in line with this topographic organization of the putamen. RHw recruits the anterior associative territories more than the other tasks performed with any of the other limbs, even though it is more difficult to write with the left non-dominant hand or the foot [54], [55]. Thus, we think that the anterior part of the putamen is recruited for integrating a cognitive goal-oriented task and dexterous motor control, which are required for writing with the dominant hand.

### Limitations

Finding an optimal control task for writing is difficult since this task involves motor control, sensory feedback, memory, and language. Other studies have implemented different control tasks, focusing on movement complexity [5], the language component [5], [20], right versus left limb [9], and meaningful versus meaningless graphemes. We chose to control for the language/memory aspects of the task by having the subjects write with the LH and the RF; we controlled for right-hand movement by having subjects perform tapping and zigzagging with their right hands. We acknowledge that the zigzagging task requires less dexterity than RHw. Thus, it is highly possible that the network supporting task specificity would be activated for other highly skilled and dexterous motor tasks. Our results help increase the knowledge of spatial organization within the premotor and parietal cortices and illustrate potential regions of interest that warrant future studies.

While we did not measure proficiency in writing *per se*, all subjects had at least 12 years of education at the time of their scans. It remains to be determined whether the premotor-parietal-cerebellar regions we found uniquely activated for RHw are similar to those active for a different over-learned task. Further studies would have to test the existence of brain networks that are specifically recruited during other over-learned tasks; for example, professional musicians playing a well-known piece of music with the instrument in which they are specialists versus with an instrument they are not proficient. Studying RHw as a model for task specificity, however, provides better understanding of the physiology of a complex and over-learned movement, and sets the basis for further studies in writer’s cramp patients.
